# Investigating the Impact of Fast‐Paced Digital Content, Social Media Engagement, and Lifestyle Factors on Attention, Cognitive Load, and Behavioral Impulsivity: A Machine Learning Approach

**DOI:** 10.1002/brb3.71651

**Published:** 2026-08-12

**Authors:** Arfatul Islam Asif, Sanjoy Das Joy, Mustakim Billah, Unayes Ahmed Khan, Ibnul Mansib, Sheikh Md. Azizul Hoque Munna, Anupam Kumar Bairagi, Abdullah Al Noman

**Affiliations:** ^1^ Department of Computer Science & Engineering Shahjalal University of Science and Technology Sylhet Bangladesh; ^2^ Computer Science & Engineering Discipline Khulna University Khulna Bangladesh

**Keywords:** attention, cognitive load, digital well‐being, impulsivity, machine learning

## Abstract

**Background:**

The increase in fast‐paced digital content and changes in lifestyle among young adults have raised concerns about their effects on cognitive functions. While many studies have looked into this connection, few have used strong machine learning models to predict subclinical cognitive outcomes based on real‐world, self‐reported behaviors.

**Objective:**

This study aimed to develop and evaluate machine learning models that predict levels of attention, cognitive load, and behavioral impulsivity using self‐reported digital usage and lifestyle factors.

**Methodology:**

A cross‐sectional study included 301 participants who filled out a custom 55‐item online questionnaire. Four machine learning algorithms (K‐nearest neighbors [KNN], support vector regression [SVR], random forest, extreme gradient boosting [XGBoost]) were evaluated for each of the three cognitive‐behavioral outcomes using a 5 × 5 nested cross‐validation for unbiased evaluation. Feature importance was established using permutation importance and confirmed with SHapley Additive exPlanations (SHAP).

**Results:**

The models showed strong predictive power, especially for attention, where the random forest model had the best performance (*R* ^2^ = 0.513 ± 0.075). SVR was most effective for cognitive load, while XGBoost was the best for behavioral impulsivity. A key finding from the feature importance analysis was that technology usage habits were the main predictors for attention. In contrast, lifestyle factors were the strongest predictors for both cognitive load and impulsivity.

**Conclusion:**

Machine learning models can reliably predict cognitive and behavioral outcomes from easily accessible self‐reported data. Findings indicate that inattention is most strongly associated with digital usage behaviors, whereas cognitive load and impulsivity are more strongly associated with indicators of underlying emotional distress; because the design is cross‐sectional, these relationships are predictive rather than causal.

## Graphical abstract

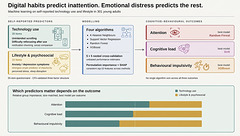

## 1 Introduction

Fast‐paced digital content (content that has a short appearance and has immediate rewards on memory, like YouTube shorts, TikTok, Facebook reels), along with constant social media usage, notification‐driven multitasking, and irregular sleep patterns, is changing how young adults maintain attention, handle cognitive load, and control impulses. The modern information and technology‐based environment, marked by constant connectivity and platforms like YouTube, TikTok, and Facebook optimized by algorithms, puts extreme pressure on our limited cognitive resources. This constant stream of stimuli is connected to a state of “continuous partial attention.” This can harm deep work, academic performance, and overall well‐being. University students are especially at risk. They face intense academic pressures while being surrounded by a digital environment that aims to capture and profit from their attention.

While research increasingly shows the negative links between screen time and cognitive outcomes, there are still important gaps. Much of the current work is descriptive, relies on single‐factor analyses, or takes place in controlled lab settings that may not capture the complexity of real‐world digital habits. Additionally, when machine learning (ML) has been used in related areas, it has mainly concentrated on predicting clinical diagnoses like anxiety, depression, or ADHD. It has not focused on the subclinical cognitive and behavioral changes that impact a wider population (Chowdhury et al. 2024; Chung and Teo 2022; Priya et al. 2020; Vaishnavi et al. 2022; Nemesure et al. 2021). Also, many earlier studies have looked at the connections between digital media and cognition; they often do not relate strongly to established cognitive theories. They also seldom use solid predictive modeling in real‐life situations. This study fills that gap by using a theory‐based ML approach. It examines how daily digital use and lifestyle habits predict important cognitive and behavioral outcomes.

To understand the potential mechanism behind these real‐world observations, this investigation follows several theoretical frameworks. It draws on the Capacity Theory of Attention (Kahneman 1973) (which states that focus is a limited mental resource) and the Cognitive Load Theory (Sweller 2011) (which considers working memory a finite system). This research puts these concepts into action by measuring the effects of multitasking driven by notifications and other digital distractions that create extraneous load. The study also uses dual‐process models (Kahneman 2011), which argue that cognition operates through two competing systems: a fast, automatic, and intuitive system (System 1) driven by impulse, and a slow, effortful, and logical system (System 2) responsible for deliberate self‐control. This framework is tested by looking at how digital cues, such as the urge to check notifications, trigger impulsive System 1 behaviors that ignore the deliberate goals managed by System 2. Finally, the investigation combines Self‐Regulation Theory, which sees willpower as a resource that can run out (Baumeister et al. 1998), with Compensatory Internet Use Theory, which suggests people turn to media to deal with negative feelings (Kardefelt‐Winther 2014). This is examined by measuring how lifestyle factors like stress and poor sleep, alongside behaviors like using social media to escape, predict cognitive outcomes. The predictive ability of behaviors linked to these mechanisms is then quantified using ML models built from everyday digital and lifestyle habits. Although these outcomes—inattention, cognitive load, and impulsivity—are related and frequently co‐occur, they are treated here as conceptually distinct constructs with somewhat different causes; emotional distress, in particular, is modeled as a predictor of these outcomes rather than as an outcome itself (see Section 3.1.4).

This study makes several novel contributions to the literature. Methodologically, it goes beyond traditional correlation techniques. It uses a rigorous 5 × 5 nested cross‐validation procedure to ensure unbiased model performance estimation. It also applies a size‐matched permutation importance analysis for a fair comparison between feature groups. Unlike studies that rely on lab‐based or physiological data, this research shows the predictive power of accessible, self‐reported data. This highlights a scalable approach for future digital well‐being tools. Conceptually, the study's unique contribution lies in its effort to ground the predictive model in established psychological theory. By applying concepts from various integrated frameworks such as Capacity Theory of Attention, Cognitive Load Theory, Dual‐Process Models, Self‐Regulation Theory, and Compensatory Internet Use Theory, the research goes beyond simple pattern detection to create a clear, data‐driven narrative that is interpreted in light of the potential mechanisms linking digital habits to cognitive outcomes. The research addresses the following questions:

- RQ1: To what extent can an individual's daily technology usage habits and their lifestyle factors predict their levels of inattention, cognitive load, and behavioral impulsivity?
- RQ2: What is the relative importance of technology usage versus lifestyle factors in predicting these negative cognitive and behavioral outcomes?
- RQ3: Which specific behaviors, such as social media usage, multitasking, or sleep patterns, have the most significant impact on these outcomes, providing insight into potential mechanisms?
- RQ4: Which machine learning algorithm (e.g., support vector regression [SVR], extreme gradient boosting [XGBoost]) proves most effective and robust for modeling the complex relationship between digital habits, lifestyle, and cognitive well‐being?

The rest of this paper is organized as follows. Section 2 reviews the related literature. Section 3 explains the methodology, including data collection and the predictive modeling pipeline. Section 4 presents the results and answers the research questions. Section 5 discusses the theoretical and practical implications of the findings. Section 6 wraps up the study and suggests directions for future research.

## 2 Literature Review

### 2.1 The Cognitive and Behavioral Impact of Digital Technology Usage

The constant use of digital technology in everyday life has led to significant research on its impact on cognitive functions and behaviors, including attention, focus, cognitive load, and impulsivity. Many related studies suggest that regular exposure to fast‐paced digital content, social media use, and multitasking are closely related to reduced attention, increased cognitive load, and higher impulsivity. These findings show that using technology, especially multitasking with social media while performing academic tasks, often harms performance due to poor attention control. Increased technology usage and social media multitasking are not only linked to lower academic success but also to a diminished ability to filter out irrelevant information and maintain focus (Kokoç 2021; Swain and Pati 2019; May and Elder 2018; Shanmugasundaram and Tamilarasu 2023; Junco and Cotten 2012).

The features of modern digital media, especially short‐form videos, seem to make these problems worse. This content has a fast pace and a high‐arousal feel. It might strengthen a desire for instant rewards, which can weaken the ability to stay focused on complex tasks (Asif and Kazi 2024; Qin et al. 2022; Yan et al. 2024). Research has shown a strong link between watching short videos and an increase in inattentive symptoms. This effect is especially noticeable in younger people, as their executive functions are still developing (Chiencharoenthanakij et al. 2025). Studies go as far as showing that students can focus for only approximately 6 min in the presence of technological distractors such as texting and social media updates (Rosen et al. 2013).

This phenomenon of “continuous partial attention” can reduce productivity, impair memory retention, and increase stress. Moreover, researchers have seen a negative link between high social media engagement and self‐control over rewards. This suggests that the instant feedback from online platforms may weaken self‐control and encourage impulsive behavior (Shanmugasundaram and Tamilarasu 2023; Meshi et al. 2019). The constant switching between tasks in digital environments comes with a cognitive “reorientation cost.” This cost drains limited mental resources, such as working memory (Subardjo and Khan 2025; Ophir et al. 2009; Strayer and Johnston 2001).

### 2.2 AI and ML in Mental Health Prediction

ML and AI have become effective methods for prediction and analysis in psychological and behavioral science. ML algorithms are good at finding complex, nonlinear patterns in large datasets. This makes them suitable for modeling the many factors involved in mental health. A review of the field shows that random forest (RF) and support vector machine (SVM) are among the most commonly used models for predicting various conditions, including depression, anxiety, schizophrenia, and PTSD (Chung and Teo 2022). These models have been successfully deployed on diverse data sources, from clinical electronic health records (EHRs) and survey responses to behavioral data from social media and speech patterns.

Many recent studies highlight the use of ML in identifying at‐risk populations. One study on Bangladeshi students found alarmingly high rates of anxiety (83.3%), depression (84.7%), and insomnia (76.5%) among this group. XGBoost was the most effective prediction model, based on factors like social media addiction and academic performance (Chowdhury et al. 2024). Another study created a new hybrid depression assessment scale. It achieved a 98.08% prediction accuracy using an RF model on a detailed dataset of student lifestyle and psychosocial factors (Siddiqua et al. 2023). Beyond survey data, researchers have effectively used nonpsychiatric EHR data to predict major depressive disorder and generalized anxiety disorder. This shows the potential for early and low‐burden screening (Nemesure et al. 2021).

Despite these successes, the literature also points out ongoing challenges. These include small sample sizes and the urgent need for better model interpretability and external validation. This is essential to ensure clinical applicability (Chung and Teo 2022).

### 2.3 ML Approaches for Specific Cognitive Traits Related to This Study

While ML has been widely used for general mental health disorders such as depression, anxiety, ADHD, and insomnia, its role in predicting specific cognitive and behavioral traits central to this research, such as attention, cognitive load, and impulsivity, is more specialized. Much of the work done in this area has focused on studying physiological and detailed behavioral data collected during controlled tasks instead of relying on self‐reported, real‐world habits. For example, several studies have successfully predicted a person's level of visual attention by using ML models on data from eye trackers. These methods examine factors like gaze patterns, head movements, and reading time to classify focus into categories like “High,” “Average,” or “Low” (Chakraborty et al. 2020). Similarly, research on cognitive load has made extensive use of gaze‐tracking technology to offer real‐time insights into a learner's mental state. By looking at metrics such as fixation duration and eye movement patterns, ML models can predict cognitive overload, often in specific contexts like eLearning environments (Ajayi 2024).

These sensor‐based methods provide high precision, but they differ greatly from the approach in this study. While these studies support the use of ML for modeling our target outcomes, they mainly rely on specialized equipment and specific data. There is still a gap in understanding how well these cognitive and behavioral traits can be predicted using more accessible, self‐reported data about everyday technology use and lifestyle factors. This is the main goal of this investigation.

A summary of the literature review section across three key domains is presented in Table 1.

**TABLE 1:** Summary of literature: digital habits, cognitive impacts, and predictive modeling.

Reference	Research focus	Sample and data source	Methods and algorithms	Key limitations
**Panel A: Digital technology's cognitive and behavioral impact**				
(Kokoç 2021)	Social‐media multitasking versus attention and academic outcomes	*N* = 637 students; paper survey and GPA	Mediation modeling	Cross‐sectional; self‐reported measures
(Chiencharoenthanakij et al. 2025)	Short‐form video (SFV) links to ADHD and inattentive behaviors	*N* = 528 children; hospital parent survey	Statistical correlation	Cross‐sectional; parent‐report bias
(Subardjo and Khan 2025)	Digital multitasking effects on cognitive load and attention	N/A (literature review)	Conceptual framework/narrative analysis	No primary data; narrative synthesis
(Asif and Kazi 2024)	SFV use versus attention difficulties and academic performance	*n* = 210 students; survey and interviews	Mixed methods	Purposive sample; lacks objective tracking
(Shanmugasundaram and Tamilarasu 2023)	Tech/AI impact on cognition, reward, and decision‐making	N/A (literature review)	Narrative synthesis	Not systematic; mostly correlational
(Hayes et al. 2025)	Fast‐paced/violent media versus ADHD and impulsivity	*N* = 1127 students (three studies); online surveys	Correlational analysis	Cross‐sectional; confounding variables
**Panel B: Machine learning in mental health prediction**				
(Chung and Teo 2022)	ML for mental health prediction (e.g., depression, PTSD)	30 studies (systematic review)	SVM, RF, DL, ensembles	Small samples; limited external validation
(Priya et al. 2020)	DASS‐21 severity level empirical prediction	*n* = 348 adults; online survey	DT, RF, NB, SVM, KNN	Imbalanced classes; internal split only
(Vaishnavi et al. 2022)	Survey‐based mental health illness prediction	*n* = 1259 records; survey	LR, KNN, DT, RF, stacking	Single dataset; lacks external validation
(Nemesure et al. 2021)	MDD and GAD prediction from general EHR data	*n* = 4184 undergraduates; EHR	Ensembles (XGBoost), SHAP	Single institution; low disorder prevalence
(Siddiqua et al. 2023)	Hybrid depression assessment for students	*n* = 594 students; mixed‐mode survey	10 ML models + ANN/CNN, LIME	High questionnaire burden; engineered label
(Chowdhury et al. 2024)	Anxiety, depression, insomnia risk factors in students	*n* = 1250 students; social media survey	DT, RF, XGBoost	Convenience sample; tree‐based models only
**Panel C: Machine learning approaches for specific cognitive traits**				
(Chakraborty et al. 2020)	Attention level (high/avg/low) prediction from behavioral signals	*N* = 400 students; survey and eye‐tracking	LR, SVM, DT, KNN, AdaBoost, MLP	Single cohort; partial self‐reported inputs
(Ajayi 2024)	Cognitive load prediction in e‐learning environments	Simulated gaze‐tracking dataset	LR, RF, SVM (RBF)	Simulated data requires real‐world validation

## 3 Methodology

### 3.1 Questionnaire Creation

The questionnaire for this study was crafted to collect a detailed set of predictor variables (digital habits and lifestyle factors) and outcome variables (attention, cognitive load, and impulsivity). Drawing from established psychological theories and validated scales, the questionnaire included items adapted from existing instruments along with a few custom items designed to measure behaviors specific to modern digital environments.

#### 3.1.1 Measures

Outcome variables: Three composite scores were created as the dependent variables for the predictive models.

- Attention and Focus Scale (10 items): This scale, adapted from tools like the Adult ADHD Self‐Report Scale (ASRS v1.1) (Kessler et al. 2005), Conners’ Adult ADHD Rating Scales (CAARS) (Conners et al. 1999), and the Mindful Attention Awareness Scale (MAAS) (Brown and Ryan 2003), measured self‐reported lapses in attention and the ability to focus. The scale is given in Part C of Table A1.
- Cognitive Load Scale (10 items): Based on the Multidimensional Fatigue Inventory (MFI‐20) (Smets et al. 1995) and the Fatigue Assessment Scale (FAS) (Michielsen et al. 2004), this scale assessed feelings of mental exhaustion from cognitive effort. The scale is given in Part D of Table A1.
- Behavioral Impulsivity Scale (10 items): Adapted from the Barratt Impulsiveness Scale (BIS‐11) (Patton et al. 1995) and the Perceived Stress Scale (PSS) (Cohen et al. 1983), this scale measured the tendency to act on impulse without sufficient forethought. The scale is given in Part E of Table A1.

Internal consistency for each outcome scale was assessed after data collection using Cronbach's *𝛼* (Cronbach 1951) and McDonald's *ω* (McDonald 1999), and its structural and discriminant validity was further examined through confirmatory factor analysis (CFA), reported in Section 3.3.5

Predictor variables: A set of 25 predictor variables was created to capture a diverse range of daily behaviors and psychosocial states relevant to cognitive function. The choice of these items was guided by established psychological theories to cover factors known to affect cognition and self‐regulation. The variables fell into the following categories:

- Tech usage habits (15 items): These items aimed to quantify patterns of digital media use. They assessed behaviors related to cognitive overload and attention‐switching costs (e.g., Q8, distractions from notifications; Q15, challenges refocusing after use), cue‐driven impulsivity (e.g., Q3, urge to check notifications; Q7, anxiety over post social media engagement), and failures of self‐regulation (e.g., Q2, unintended scrolling). The tech usage habits questionnaire items are detailed in Part A of Table A1.
- Lifestyle and psychosocial factors (10 items): These items measured important personal factors that can affect cognitive resources and emotional well‐being. This included measures assessing the state of regulatory resources (e.g., Q17, sleep disturbances; Q20, stress levels) and identifying emotional drivers, such as anxiety (Q21), that often lead to compensatory digital behavior. The lifestyle and psychosocial factors questionnaire items are provided in Part B of Table A1.

Demographics: Basic demographic information, specifically age and gender, was also collected as predictor variables.

#### 3.1.2 Scoring and Format

All questionnaire items uniformly used a five‐point Likert scale, ranging from 1 (strongly disagree) to 5 (strongly agree). Reverse‐scored items were appropriately adjusted during preprocessing to ensure consistent interpretation across the composite scores.

#### 3.1.3 Questionnaire Validation and Evaluation

Content validity was established prior to data collection through a two‐step expert‐and‐pilot review. The full item set was evaluated by a psychologist at Shahjalal University of Science and Technology to confirm that each item was relevant, clearly worded, and representative of its intended construct. The revised questionnaire was then pilot‐tested with 10 participants, whose feedback on clarity and interpretation was incorporated into the final set of 55 items. The structural validity of the resulting scales was subsequently examined empirically via CFA (Section 3.3.5).

#### 3.1.4 Construct Definitions

The three outcome constructs are defined as follows. Inattention is the failure to initiate or sustain controlled, goal‐directed focus (operationalized via ASRS/CAARS/MAAS‐derived items). Cognitive load is the subjective experience of mental effort and fatigue arising from demands on limited working‐memory resources (operationalized via MFI‐20/FAS‐derived items). Behavioral impulsivity is the tendency to act without enough forethought or control (operationalized via BIS‐11/PSS‐derived items). Emotional distress (anxiety, depression, and stress; Q20, Q21, Q24) is modeled not as an outcome but as a predictor within the lifestyle and psychosocial block, representing an antecedent state hypothesized to deplete the regulatory resources that govern the three outcomes.

### 3.2 Data Collection

**FIGURE 1:**
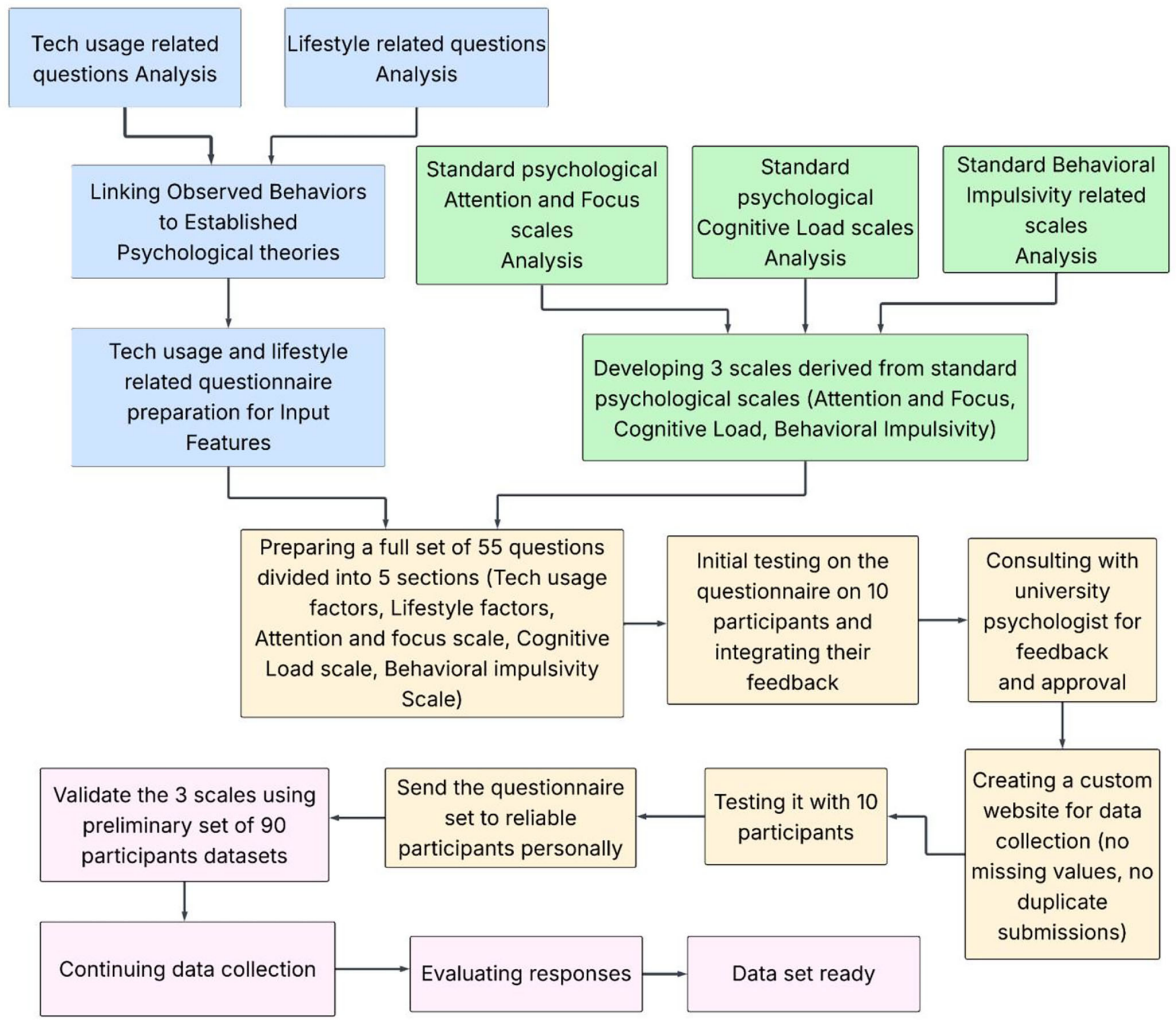
Study design and data collection process.

Data were collected between June 2025 and August 2025 through a custom‐built web application specifically designed for this study. The application presented the questionnaire in five distinct sections: tech usage, lifestyle factors, attention and focus scale, cognitive load, and behavioral impulsivity. This structure ensured clarity for participants. A key feature of the system required all questions to be answered, which prevented missing data and simplified the preprocessing pipeline by removing the need for data imputation. Also, the system was designed to make unintentional duplicate submissions impossible. It should be noted that the tech‐usage items captured general patterns of engagement (e.g., unintended scrolling, notification checking, difficulty refocusing after use) rather than logged exposure to specific short‐form‐video platforms. The study design and data collection process are illustrated in Figure 1.

#### 3.2.1 Participant Recruitment and Consent

Participation in the study was voluntary, and anyone willing to complete the entire questionnaire could access the survey. Before starting, participants saw a digital informed consent form. This form outlined the study's purpose and basic information about data use, privacy, and anonymity. Individuals who agreed proceeded with the questionnaire. The questionnaire was mainly given to university students in Bangladesh. It also included a specific sample of college students and young adults, along with some individuals in their late 30s.

#### 3.2.2 Scale Reliability

A preliminary (pilot) reliability check was conducted on an initial sample of 90 participants to confirm the internal consistency of the three outcome scales (attention, cognitive load, and behavioral impulsivity) before expanding data collection. Both Cronbach's *𝛼* and McDonald's *ω* exceeded the standard threshold of 0.70 for all three scales (reported in Table 2). Data collection was then expanded to the final sample, and internal consistency was subsequently reconfirmed on the full sample via the composite reliability estimates obtained from the CFA (Section 3.3.5), which closely reproduced these pilot values.

**TABLE 2:** Reliability of outcome scales: Cronbach's alpha (*α*) and McDonald's omega (*ω*).

Outcome scale	Cronbach's alpha (*α*)	McDonald's omega (*ω*)
Attention	0.859	0.860
Cognitive load	0.839	0.849
Behavioral impulsivity	0.710	0.724

### 3.3 Data Exploration

#### 3.3.1 Descriptive Statistics

The final dataset included responses from 301 participants, including 176 males (58.5%) and 125 females (41.5%). The age distribution of the sample is as follows:

- Female participants were mostly between the ages of 20 and 23, with the largest group at age 23 (29 participants), followed by age 21 (22 participants) and age 22 (19 participants). The age range was broad, including some respondents in their mid‐to‐late teens and several outliers in their late 20s and older.
- Male participants also showed a strong concentration in the early 20s, peaking at age 23 (43 participants) and 24 (36 participants). A significant number of responses also came from participants aged 19–22. Some participants were in their late 20s and mid‐30s.

Overall, the dataset represents a primarily young adult population, with the vast majority of participants aged 19–24.

#### 3.3.2 Scale Reliability

To check the internal consistency of the outcome measures, a pilot reliability analysis was performed on the first 90 participants. Both Cronbach's alpha (*𝛼*) and McDonald's *ω* values were calculated. As shown in Table 2, all three scales showed good to excellent internal consistency, with all values surpassing the standard level of 0.70. As mentioned in Section 3.3.5, these values were confirmed by the composite reliability estimates from the full‐sample CFA.

#### 3.3.3 Summary Statistics for Core Constructs

To understand the distribution of the main constructs—technology usage, lifestyle, attention, cognitive load, and behavioral impulsivity—standard measures of central tendency and dispersion were calculated for the full sample of 301 participants. The results for central tendency (mean, median, and mode) are presented in Table 3, while the measures of spread (standard deviation [SD], variance, skewness, and kurtosis) are detailed in Table 4.

- SD ranged from 0.47 to 0.72, with attention showing the highest variability among participants.
- Cognitive load had a noticeable negative skew (−0.80), indicating that more participants reported higher levels of cognitive load. The other constructs were nearly symmetric.
- Most constructs had platykurtic (relatively flat) distributions, while cognitive load was leptokurtic (kurtosis ≈ 1.68), indicating a more peaked distribution with heavier tails.

**TABLE 3:** Central tendency measures: mean, median, and mode for each category.

Category	Mean	Median	Mode
Tech usage	3.356	3.333	3.40
Lifestyle	3.150	3.100	3.10
Attention	3.320	3.300	3.20
Cognitive load	3.514	3.600	3.70
Behavioral impulsivity	3.098	3.100	3.40

**TABLE 4:** Measures of spread: standard deviation, variance, skewness, and kurtosis for each category.

Statistic	Tech usage	Lifestyle	Attention	Cognitive load	Behavioral impulsivity
Standard deviation	0.617	0.474	0.723	0.504	0.541
Variance	0.381	0.225	0.522	0.254	0.293
Skewness	−0.129	0.066	0.031	−0.796	0.254
Kurtosis	−0.204	−0.401	−0.359	1.677	−0.088

#### 3.3.4 Visualizations

**FIGURE 2:**
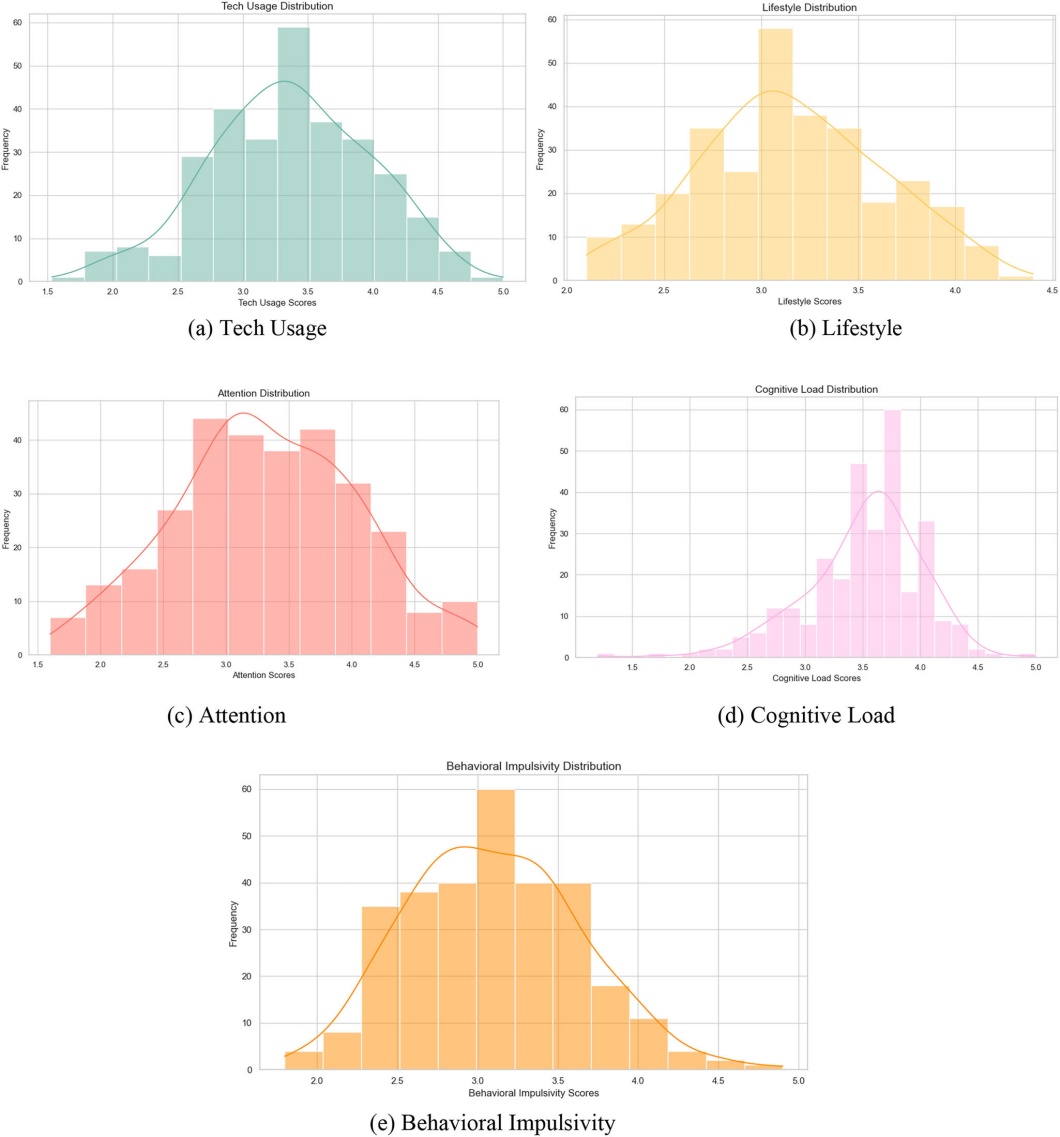
Distribution of participant scores across all core constructs: (a) tech usage, (b) lifestyle, (c) attention, (d) cognitive load, and (e) behavioral impulsivity.

The distributions of participant scores for tech usage, lifestyle, attention, cognitive load, and behavioral impulsivity are illustrated in Figure 2a–e.

#### 3.3.5 Construct Validity (CFA)

A CFA was conducted on the full sample (*N* = 301) using robust maximum likelihood estimation (Satorra and Bentler 2001), with the 30‐outcome items loading onto their three respective latent factors. Absolute fit was acceptable (*χ* ^2^/df = 2.14; RMSEA = 0.062; SRMR = 0.065, both within the ≤ 0.08 threshold), while incremental indices fell slightly below the 0.90 cutoff (CFI = 0.839; TLI = 0.826) (Hu and Bentler 1999)—a pattern common for scales adapted from multiple source instruments. Composite reliability was strong for all three factors (0.851/0.833/0.734), reproducing the *α* and *ω* values in Table 4 on the full sample.

To confirm the outcomes were distinguishable rather than a single dimension, the three‐factor model was compared against a one‐factor model; it fit significantly better (Δ*χ* ^2^(3) = 221.9, *p* < 0.001; ΔCFI = +0.077) (Table 5). Latent inter‐factor correlations (0.69–0.79) remained below the 0.85 redundancy threshold (Fornell and Larcker 1981), and observed composite‐score correlations were lower still (0.59–0.69), indicating that the three constructs are strongly related yet statistically distinct.

**TABLE 5:** Confirmatory factor analysis: fit indices and one‐factor versus three‐factor model comparison (*N* = 301).

Model	*χ* ^2^	df	*χ* ^2^/df	CFI	TLI	RMSEA	SRMR
One‐factor	1081.9	405	2.67	0.762	0.745	0.075	—
Three‐factor	860.0	402	2.14	0.839	0.826	0.062	0.065
Difference (3‐ vs. 1‐factor)	Δ*χ* ^2^(3) = 221.9, *p* < 0.001			ΔCFI = +0.077			

*Note*: Estimation used robust maximum likelihood. AIC/BIC are not reported for this nested comparison, as the likelihood‐ratio (*χ* ^2^‐difference) test is the appropriate criterion.

One caveat is noted and revisited in Section 5.4. Average variance extracted fell below the 0.50 benchmark (0.37/0.34/0.26), reflecting the breadth of the source instruments (notably the multidimensional BIS‐11) and weaker loadings on the negatively‐worded items (Q41, Q44, Q55), a known method effect (Woods 2006); convergent validity nonetheless remains adequate given composite reliability above 0.60 (Fornell and Larcker 1981).

### 3.4 Predictive Modeling

The predictive modeling for this study used a rigorous, multi‐stage method to ensure unbiased performance evaluation, robust feature importance analysis, and transparent model interpretation. Each of the four ML algorithms—K‐nearest neighbors (KNN) (Cover and Hart 1967), SVR (Vapnik et al. 1996), RF (Breiman 2001), and XGBoost (Chen and Guestrin 2016)—followed the same pipeline from preprocessing to interpretation. All analyses were done using Python with the scikit‐learn (Pedregosa et al. 2011) and SHapley Additive exPlanations (SHAP) libraries (Lundberg and Lee 2017).

#### 3.4.1 Data Preparation and Preprocessing

Before training the model, the data went through several preprocessing steps. First, unnecessary columns from the data export, like _id and email, were removed. The age variable was cleaned to ensure it was numeric. All specified reverse‐scored items were inverted—for instance, a score of 1 became 5.

The three outcome variables—attention, cognitive load, and behavioral impulsivity—were created by calculating the mean score of their respective 10‐item scales. The final set of predictor variables included 27 features: the 25 questionnaire items (Q1–Q25), age, and gender.

To avoid data leakage and prepare the data for the algorithms, a preprocessing pipeline was set up using scikit‐learn. This pipeline used a ColumnTransformer to apply StandardScaler to all numeric features (Q1–Q25, age) and OneHotEncoder to convert the categorical gender feature into a numeric format. This entire preprocessing pipeline was integrated into the cross‐validation process. This ensured that scaling parameters were learned only from the training data in each fold.

#### 3.4.2 Unbiased Performance Evaluation Using Nested Cross‐Validation

To avoid optimistic bias and get a realistic estimate of how each model would perform on new, unseen data, a 5 × 5 nested cross‐validation procedure (Varma and Simon 2006) was used. This method separates hyperparameter tuning from the final model performance evaluation.

- Outer loop (five folds): The dataset was divided into five outer folds. In each iteration, one fold was set aside as the final test set, simulating a “real‐world” test. The remaining four folds were used for model training.
- Inner loop (five folds): Within each outer loop, a fivefold inner cross‐validation was done on the outer training data. A GridSearchCV helped find the optimal hyperparameters for the model from a predefined search space.

The best model from the inner loop was then tested on the held‐out outer test set. Performance was recorded using three standard metrics: *R* ^2^, root mean squared error (RMSE), and mean absolute error (MAE). This process was repeated for all five outer folds, and the resulting metrics were averaged to calculate their mean and ±SD. The formulas and interpretations for the evaluation metrics used in this study are detailed in Table 6.

**TABLE 6:** Evaluation metrics for predictive models: formulas and interpretations.

**Metric**	**Formula**	**Interpretation**
$R^{2}$	$1\;-\;\frac{\sum_{i=1}^{n}{\left(y_{i}\;-\hat{\;y}i\right)}^{2}}{\sum_{i=1}^{n}{\left(y_{i\;}-\;\bar{y}\right)}^{2}}$	The proportion of variance in the outcome that the model can predict. Higher is better.
**RMSE**	$\sqrt{\frac{1}{n}\sum_{i=1}^{n}{\left(y_{i}-\hat{y_{i}}\right)}^{2}}$	The average size of the prediction error, in the original units of the outcome. Lower is better.
**MAE**	$\frac{1}{n}\sum_{i=1}^{n}\left|y_{i}-\hat{y_{i}}\right|$	The average absolute difference between the predicted and actual values. Lower is better.

#### 3.4.3 Feature Importance Analysis

To find out which predictors were most influential, permutation importance (Breiman 2001) was calculated. This technique measures a feature's importance by observing how much the model's performance (*R* ^2^) decreases when that feature's values are randomly shuffled. A large drop in performance shows an important feature. This analysis was done on the outer test set within each of the five outer folds to ensure the importance estimates were unbiased.

- Individual feature importance: The importance of each predictor was calculated by measuring the mean decrease in the model's *R* ^2^ score after shuffling that feature's values 30 times. The final importance score was reported as the mean drop across all five outer folds, along with a 95% confidence interval (CI).
- Group importance and size‐matching: To make a fair comparison between the tech usage (15 features) and lifestyle (10 features) groups, a size‐matched analysis was carried out. Note that 200 random subsets of 10 features were drawn from the tech usage group, and their mean importance was calculated, and this was used as group importance for the tech usage group. The final reported percentages were based on the contributions of the tech usage group compared to the lifestyle group.

In this predictive framework, a feature's importance reflects the amount of unique predictive information it carries about an outcome rather than a causal effect on that outcome. Correspondingly, a SHAP value indicates the direction and magnitude with which a feature's value pushes a given prediction higher or lower. The importance rankings reported below should therefore be read as identifying which behaviors are most informative about an outcome, not which behaviors cause it.

#### 3.4.4 Final Model Selection and Interpretation

While the nested cross‐validation provided an unbiased performance estimate, a separate process was used to select and train a single final model for interpretation.

- Final hyperparameter selection: For each outcome, a fivefold cross‐validated GridSearchCV was run on the entire dataset to find the best hyperparameter set for the final model.
- Final model refitting: Using the best hyperparameters found, a final model was trained one last time on the entire dataset.
- Model interpretation with SHAP: To understand how the final model made its predictions, SHAP values were computed. SHAP is a method based on game theory that explains the prediction for an individual case by assigning an importance value to each feature, which shows its role in pushing the prediction higher or lower. To verify the findings, the top 10 features identified by SHAP were compared to the top 10 from the unbiased permutation analysis, and the consistency was reported as a percentage match.

**FIGURE 3:**
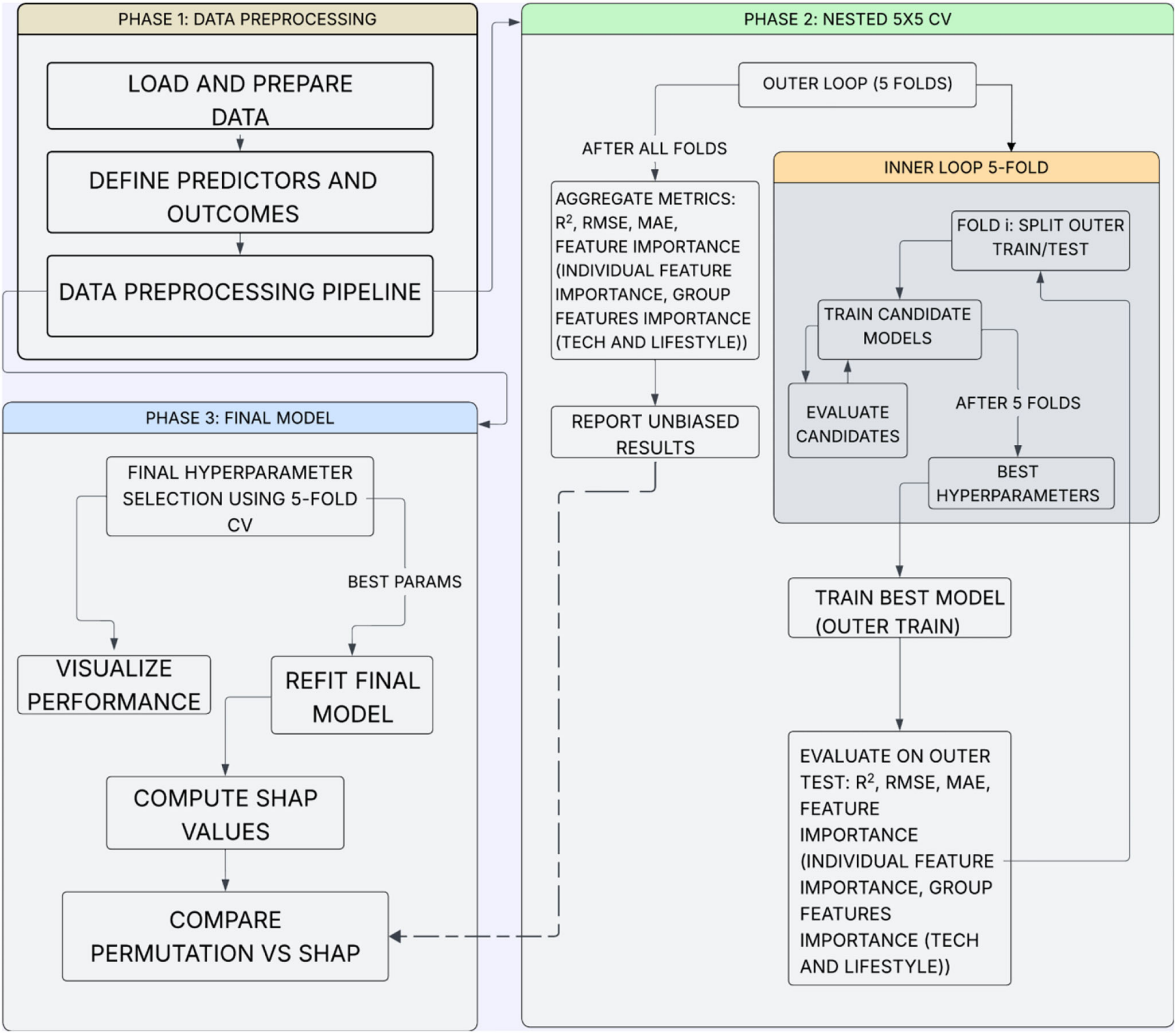
End‐to‐end predictive modeling pipeline: preprocessing, nested CV, permutation importance, final refit, and SHAP interpretation.

The complete sequence of these final steps, alongside the preceding validation phases, is illustrated in the end‐to‐end pipeline in Figure 3.

#### 3.4.5 RF

RF is an ensemble learning method that builds many decision trees during training. For regression tasks, the final prediction comes from averaging the predictions of the individual trees. This approach reduces the tendency of single decision trees to overfit, often resulting in a more accurate and stable model.

Methodology: The RF model was optimized using the GridSearchCV process. The hyperparameter grid explored a range of model complexities appropriate for the dataset size. The search space included key hyperparameters such as n_estimators (the number of trees in the forest; tested values: [50, 100, 200, 300, 500]), max_depth (the maximum depth of each tree; [None, 6, 10, 14, 18]), min_samples_split [2, 4, 8, 12], min_samples_leaf [1, 3], and max_features (the number of features to consider for the best split; [“sqrt,” 0.5]).

##### 3.4.5.1 Final Model Selection and SHAP Interpretation

The best hyperparameters for the final refit models were chosen using a fivefold cross‐validated grid search on the entire dataset. The results are shown in Table A2, along with a feature importance analysis using SHAP.

SHAP interpretation: The SHAP analysis of the final refit models mostly confirmed the findings from the permutation importance analysis. The top 10 features identified by SHAP were compared with the top 10 from the unbiased nested CV permutation analysis, showing the following consistency:

- Attention: 9/10 (90% agreement)
- Cognitive load: 9/10 (90% agreement)
- Behavioral impulsivity: 9/10 (90% agreement)

This strong agreement indicates that the identified feature importances are reliable across different evaluation methods.

#### 3.4.6 KNN

KNN is a nonparametric, distance‐based algorithm. It predicts a new data point by averaging the target values of its “k” nearest neighbors in the feature space. The closeness of neighbors is usually determined by a distance metric, like Euclidean distance.

Methodology: The KNN model was optimized using the GridSearchCV process. The main hyperparameter, n_neighbors (the number of neighbors, k), was adjusted within the range of [3, 5, …, 51] to find the best value that maximized predictive performance for each outcome.

##### 3.4.6.1 Final Model Selection and SHAP Interpretation

The best hyperparameter for the final refit models was chosen through a fivefold cross‐validated grid search on the entire dataset. The results are shown in Table A3, along with an analysis of the model's feature importances using SHAP.

SHAP interpretation: The SHAP analysis of the final refit models was compared with the permutation importance analysis to confirm the stability of the findings. The consistency in the top 10 features between the two methods was as follows:

- Attention: 7/10 (70% agreement)
- Cognitive load: 5/10 (50% agreement)
- Behavioral impulsivity: 6/10 (60% agreement)

The moderate‐to‐high degree of agreement for attention and impulsivity suggests that the identified feature importances are reasonably robust. The lower agreement for cognitive load aligns with the permutation importance results, indicating that the model struggled to find a stable set of predictors for this outcome.

#### 3.4.7 SVR

SVR is a supervised learning algorithm that finds an optimal hyperplane to fit the data. Instead of minimizing error‐like traditional regression, SVR fits the error within a specific threshold or margin. Data points on the margin are called support vectors, and they play a crucial role in defining the model's fit.

Methodology: The SVR model was improved using the GridSearchCV process. The hyperparameter grid aimed to find the best combination of C (the regularization parameter that controls the penalty for misclassified data points; tested values: [0.0001, 0.01, 0.03, 0.1, 1, 10, 100]), gamma (which sets the influence of a single training example; [1e−4, …, 3]), and epsilon (which specifies the margin of tolerance where no penalty is applied to errors; [0.01, 0.1]). All models were trained using the radial basis function [rbf] kernel.

##### 3.4.7.1 Final Model Selection and SHAP Interpretation

The best hyperparameters for the final models were picked through a fivefold cross‐validated grid search on the entire dataset. The results are shown in Table A4, followed by an analysis of the model's feature importance using SHAP.

SHAP interpretation: The SHAP analysis of the final models generally supported the findings from the permutation importance analysis. The top 10 features identified by SHAP were compared to the top 10 from the unbiased nested CV permutation analysis, showing the following consistency:

- Attention: 9/10 (90% agreement)
- Cognitive load: 9/10 (90% agreement)
- Behavioral impulsivity: 9/10 (90% agreement)

This strong agreement suggests that the feature importances identified are solid and trustworthy across different evaluation methods.

#### 3.4.8 XGBoost

XGBoost is a strong and efficient version of the gradient boosting framework. It builds models one after another. Each new model fixes the errors made by the previous one. It is popular for its speed and effectiveness, using regularization to avoid overfitting and having features that work well with complex data.

Methodology: The XGBoost model was fine‐tuned using the GridSearchCV process. The hyperparameter grid was set up to test different model setups. The search space included important hyperparameters such as n_estimators (the number of boosting rounds; [50, 100, 200, 300, 500]), learning_rate (which reduces the impact of each tree; [0.01, …, 1]), max_depth (the highest depth of each tree; [2, 3, 4, 5, 6]), and subsample (the percentage of observations to be randomly sampled for each tree; [0.7, 1.0]).

##### 3.4.8.1 Final Model Selection and SHAP Interpretation

The best hyperparameters for the final, refit models were chosen using a fivefold cross‐validated grid search on the full dataset. The results are shown in Table A5, followed by an analysis of the model's feature importances using SHAP.

SHAP interpretation: The SHAP analysis of the final refit models mostly confirmed the findings from the permutation importance analysis. The top 10 features identified by SHAP were compared to the top 10 from the unbiased nested CV permutation analysis, showing considerable overlap:

- Attention: 8/10 (80% agreement)
- Cognitive load: 7/10 (70% agreement)
- Behavioral impulsivity: 10/10 (100% agreement)

The high agreement, especially for behavioral impulsivity, shows that the identified feature importances are solid and consistent across different evaluation methods.

### 3.5 Analysis Transparency

This study was not preregistered. The pilot reliability analysis of the three outcome scales (Section 3.2.2) was planned a priori, prior to full data collection. The CFA (Section 3.3.5) and the predictive modeling and feature importance analyses (Section 3.4) were conducted on the complete dataset and are exploratory in nature.

## 4 Results

### 4.1 RQ1: Predictive Power of Digital and Lifestyle Habits

The first research question aimed to find out how much daily technology use and lifestyle factors could predict levels of inattention, cognitive load, and behavioral impulsivity. The four ML models, KNN, SVR, RF, and XGBoost, were assessed using *R* ^2^, RMSE, and MAE.

The results, shown in Table 7, suggest that self‐reported tech usage and lifestyle behaviors can indeed strongly predict these cognitive and behavioral outcomes. Importantly, attention was the most predictable outcome, with the RF model achieving the highest performance (*R* ^2^ = 0.513). All models showed at least moderate predictive power for behavioral impulsivity. Performance for cognitive load was generally lower, with the SVR model showing the most promise (*R* ^2^ = 0.380), while the KNN model did not find a reliable predictor for this specific outcome.

**TABLE 7:** Unbiased performance estimates of all models across outcomes (mean ± SD).

Outcome	Model	𝑅^2^	RMSE	MAE
Attention	Random forest	**0.513 ± 0.075**	**0.500 ± 0.041**	**0.395 ± 0.028**
	XGBoost	0.497 ± 0.074	0.508 ± 0.043	0.408 ± 0.032
	SVR	0.484 ± 0.120	0.514 ± 0.066	0.411 ± 0.050
	KNN	0.471 ± 0.088	0.521 ± 0.046	0.422 ± 0.029
Cognitive load	SVR	**0.380 ± 0.166**	**0.461 ± 0.065**	**0.353 ± 0.040**
	Random forest	0.362 ± 0.152	0.469 ± 0.063	0.357 ± 0.037
	XGBoost	0.343 ± 0.202	0.473 ± 0.072	0.363 ± 0.045
	KNN	0.261 ± 0.189	0.504 ± 0.065	0.385 ± 0.034
Behavioral impulsivity	XGBoost	**0.359 ± 0.088**	**0.451 ± 0.037**	**0.354 ± 0.026**
	SVR	0.355 ± 0.098	0.452 ± 0.033	0.348 ± 0.030
	Random forest	0.344 ± 0.075	0.458 ± 0.045	0.361 ± 0.034
	KNN	0.288 ± 0.094	0.477 ± 0.049	0.370 ± 0.037

### 4.2 RQ2: Relative Importance of Tech Versus Lifestyle Factors

The second research question examined the relative importance of technology use compared to lifestyle factors in predicting outcomes. Table 8 shows the size‐normalized group importance percentages. This allows for a fair comparison of the predictive power for each feature from the tech usage (15 features) and lifestyle (10 features) groups.

**TABLE 8:** Size‐normalized group importance (%) across all models and outcomes.

Outcome	Model	Tech usage importance (%)	Lifestyle importance (%)
Attention	Random forest	**60.33**	39.67
	XGBoost	57.20	42.80
	SVR	55.52	44.48
	KNN	50.97	49.03
Cognitive load	Random forest	46.07	**53.93**
	XGBoost	45.13	**54.87**
	SVR	48.69	**51.31**
	KNN	0.00	0.00
Behavioral impulsivity	SVR	37.59	**62.41**
	XGBoost	38.86	**61.14**
	Random forest	41.06	**58.94**
	KNN	53.68	46.32

The results reveal a clear trend. Tech usage habits were consistently stronger predictors for attention, with the RF model showing the highest effect at 60.33%. On the other hand, lifestyle factors had a greater predictive role for behavioral impulsivity and cognitive load. This finding held true across all models except for KNN, which had difficulty with the cognitive load outcome.

### 4.3 RQ3: Most Impactful Behaviors

The third research question aimed to find the specific behaviors that were most predictive of each outcome. The top 10 feature importances from all four models were aggregated into a composite ranking table for each outcome, establishing a robust, cross‐model consensus on the most influential behaviors.

### 4.4 Predictors for Attention

The models consistently found that attention deficits are mostly predicted by tech usage habits associated with a loss of self‐regulation and the cognitive effects of prolonged engagement. The top 10 feature rankings across the different predictive models for attention are detailed in Table 9.

- Self‐regulation failure and attentional residue: Two predictors stood out across all models: Q2 (“I often scroll through social media feeds longer than I originally intended”) and Q15 (“After using social media, I find it difficult to focus on real‐world tasks”). Their repeated top‐two ranking shows that the main predictors of attention deficits are behaviors linked to a failure to stop using platforms and the resulting cognitive “hangover” associated with reduced focus.
- Emotional and social factors: The next group of important predictors related to the emotional and social effects of media use. Q22 (self‐esteem), Q25 (negative impact on relationships), and Q7 (anxiety over engagement) were regularly ranked as very influential. This indicates that attention issues come not only from distractions but are also closely tied to the psychosocial effects of social media.
- Habitual and distractive use: Lastly, behaviors showing habitual checking (Q3) and direct distraction (Q8) were consistently ranked as important predictors. This highlights the role of cue‐driven impulses and cognitive interruptions as correlates of reduced attentional capacity.

**TABLE 9:** Top 10 feature ranks across models for attention.

Feature description	RF rank	KNN rank	SVR rank	XGB rank
**Q2**. I often scroll through social media feeds longer than I originally intended.	1	2	1	1
Q15. After using social media, I find it difficult to focus on real‐world tasks.	2	1	2	2
Q22. Social media has affected my self‐esteem or body image.	4	3	7	3
Q25. I feel that social media or digital interactions have negatively affected my real‐life relationships.	5	5	6	5
Q7. I feel anxious when my posts do not receive likes, comments, or shares quickly.	3	4	Not ranked	4
Q3. I feel the urge to check my social media notifications as soon as they appear.	6	Not ranked	4	6
Age	Not ranked	6	3	Not ranked
Q23. I often compare myself to others based on their social media posts.	8	7	8	9
Q18. I engage in regular physical activity, such as exercise or sports.	Not ranked	9	5	8
Q8. Social media notifications often distract me from work, studies, or real‐life activities.	7	10	Not ranked	Not ranked

### 4.5 Predictors for Cognitive Load

The analysis showed that cognitive load is mainly influenced by a person's mental and emotional state. Lifestyle factors consistently have a greater effect than specific tech habits. The top 10 feature rankings across the predictive models for cognitive load are detailed in Table 10.

- Underlying mental health: The most influential predictors were consistently lifestyle factors related to emotional well‐being. Q24 (emotional instability) and Q21 (symptoms of anxiety/depression) ranked highest in three of the four models. This shows that the feeling of being mentally exhausted mainly comes from one's underlying mental health status.
- Depleted resources and compensatory use: Factors indicating a lack of resources were also strong predictors. Q17 (sleep disturbances from screens) and Q20 (high stress levels) were both significant. These items frequently co‐occur with Q12 (“I use social media as a way to escape from stress…”), a pattern consistent with a cycle in which individuals with depleted mental resources seek relief in media. It's worth mentioning that the KNN model had difficulty predicting this outcome, showing importance scores close to or below zero for most features.

**TABLE 10:** Top 10 feature ranks across models for cognitive load.

Feature description	RF rank	KNN rank	SVR rank	XGB rank
Q24. Over the past month, I have felt emotionally stable and in good mental health.	5	1	1	5
Q15. After using social media, I find it difficult to focus on real‐world tasks.	2	5	2	3
Q21. I have experienced symptoms of anxiety or depression in the past year.	1	8	3	1
Q2. I often scroll through social media feeds longer than I originally intended.	3	6	5	2
Q12. I use social media as a way to escape from stress or negative emotions.	4	Not ranked	4	4
Q7. I feel anxious when my posts do not receive likes, comments, or shares quickly.	7	9	Not ranked	6
Q17. I have trouble sleeping when I use screens before bedtime.	Not ranked	2	Not ranked	Not ranked
Q23. I often compare myself to others based on their social media posts.	10	3	Not ranked	Not ranked
Q20. I frequently experience high levels of stress due to academic, work, or personal pressures.	6	Not ranked	7	Not ranked
Q3. I feel the urge to check my social media notifications as soon as they appear.	Not ranked	Not ranked	6	7

### 4.6 Predictors for Behavioral Impulsivity

Similar to cognitive load, behavioral impulsivity was most strongly predicted by indicators of a person's emotional and mental state, pointing to a strong association between internal distress and impulsive behavior. The top 10 feature rankings across the predictive models for behavioral impulsivity are detailed in Table 11.

- Emotional distress: The results were clear. Q21 (“I have experienced symptoms of anxiety or depression…”) was the top predictor for behavioral impulsivity in all four models. This often showed a large difference. This indicates that impulsivity in this context mainly comes from emotional issues.
- Compensatory and maladaptive coping: After mental health status, predictors related to using technology to cope were also important. Q12 (“I use social media to escape stress…”) and Q15 (difficulty focusing after use) ranked highly in all models. This is consistent with a pattern in which people reporting emotional distress also report more impulsive behavior alongside technology‐based coping.
- Self‐regulation failure and social factors: Lastly, factors that measure a loss of control, like Q2 (“I often scroll longer than I originally intended”), along with those related to social comparison and validation (Q23, Q7), were also consistently recognized as key predictors across the models.

**TABLE 11:** Top 10 feature ranks across models for behavioral impulsivity.

Feature description	RF rank	KNN rank	SVR rank	XGB rank
Q21. I have experienced symptoms of anxiety or depression in the past year.	1	1	1	1
Q15. After using social media, I find it difficult to focus on real‐world tasks.	3	3	6	2
Q2. I often scroll through social media feeds longer than I originally intended.	5	5	4	3
Q23. I often compare myself to others based on their social media posts.	4	7	5	4
Q24. Over the past month, I have felt emotionally stable and in good mental health.	7	4	3	7
Q7. I feel anxious when my posts do not receive likes, comments, or shares quickly.	2	8	7	5
Q20. I frequently experience high levels of stress due to academic, work, or personal pressures.	6	Not ranked	2	6
Q12. I use social media as a way to escape from stress or negative emotions.	8	2	10	9
Q8. Social media notifications often distract me from work, studies, or real‐life activities.	Not ranked	6	Not ranked	Not ranked
Q25. I feel that social media or digital interactions have negatively affected my real‐life relationships.	10	Not ranked	Not ranked	8

### 4.7 RQ4: Most Effective and Robust Algorithm

The final research question aimed to find out which ML algorithm was best for modeling the link between digital habits, lifestyle, and cognitive well‐being. Based on the unbiased performance estimates from the nested cross‐validation, summarized in Table 7, a clear pattern emerged for each outcome.

- For attention: The RF model was the most effective and robust algorithm. It achieved the highest mean *R* ^2^ (0.513) and showed strong performance across all metrics.
- For cognitive load: While performance was generally lower for this outcome, the SVR model showed the highest predictive accuracy (*R* ^2^ = 0.380), narrowly beating the RF model.
- For behavioral impulsivity: The XGBoost model was the most effective, achieving the highest *R* ^2^ score (0.359) and showing strong performance.

In conclusion, no single algorithm stood out as the best. The results show that ensemble‐based tree models (RF, XGBoost) and kernel‐based models (SVR) are all very effective, and the best choice depends on the specific cognitive or behavioral outcome being predicted.

## 5 Discussion

### 5.1 Summary of Principal Findings

This study showed that daily digital and lifestyle habits can reliably predict key cognitive and behavioral outcomes. The main findings align with the four research questions:

First, for RQ1, the models confirmed that these behaviors significantly predict outcomes. Attention was the most predictable outcome. The RF model explained over 51% of its variance (*R* ^2^ = 0.513). All models demonstrated at least moderate predictive power for behavioral impulsivity. However, their performance on cognitive load was generally weaker, though still significant for more complex algorithms like SVR and RF.

Second, the analysis for RQ2 revealed a clear distinction in the drivers of these outcomes. By looking at each feature, tech usage habits were the main predictors for attention. In contrast, lifestyle factors, especially those linked to mental and emotional well‐being, had a greater influence on predicting both cognitive load and behavioral impulsivity.

Third, in response to RQ3, a set of consistently impactful behaviors appeared across all models. For attention, failures of self‐regulation (Q2, unintended scrolling) and cognitive after‐effects of media use (Q15, difficulty refocusing) were crucial. For both cognitive load and behavioral impulsivity, the strongest predictor was an individual's mental health status (Q21, symptoms of anxiety/depression). These outcomes were thus most strongly associated with indicators of internal distress.

Finally, for RQ4, no single algorithm was the best in every case. The results show that the best model choice depends on the outcome: RF worked best for predicting attention, SVR for cognitive load, and XGBoost for behavioral impulsivity.

### 5.2 Interpretation of Findings in the Context of Theory

Before interpreting these findings, an important constraint must be emphasized. Because the design is cross‐sectional and based on concurrent self‐report, all relationships reported below are predictive and associational rather than causal. The mechanistic pathways discussed represent theoretically plausible interpretations consistent with the observed predictive structure; they are not demonstrated causal chains, and the same associations are equally compatible with reverse or bidirectional influence (for example, preexisting attentional difficulty increasing susceptibility to compensatory media use). The theoretical framing below should therefore be read as an interpretive lens rather than as evidence of causation.

Rather than establishing mechanisms, the ML models identify which everyday behaviors are most informative about each outcome, and these patterns can be read against established psychological theories of attention, self‐regulation, and behavior. This section discusses the study's main findings in relation to these frameworks.

#### 5.2.1 Attention, Cognitive Load, and the Cost of Digital Distraction

The Capacity Theory of Attention holds that the ability to focus is a limited resource, such that rapidly shifting attention, for example, alternating between work and notifications incurs an efficiency cost. The attention findings are consistent with this account. This was most evident in the prominence of Q15 (“After using social media, I find it difficult to focus on real‐world tasks”), a top‐two predictor across all four models, which corresponds conceptually to the “attentional residue” or switching cost that persists after leaving a stimulating digital environment.

This connects with Cognitive Load Theory, which holds that working memory is limited and that unnecessary mental effort from distraction raises extraneous load. The cognitive load findings are likewise consistent with this view: Q15 was again among the top three predictors, suggesting that attentional residue has a dual character associated with both difficulty refocusing (an attention manifestation) and a sense of mental depletion (a cognitive‐load manifestation). Similarly, the prominence of Q8 (“Social media notifications often distract me from work…”) for attention is consistent with interruptions repeatedly taxing limited resources and thereby raising extraneous load.

Taken together, these patterns are consistent with a picture in which the distraction and task‐switching characteristic of heavy technology use are associated with higher extraneous load, reduced attention, and greater mental fatigue. This reading is reinforced by the group‐importance analysis (RQ2), in which tech usage habits were the stronger predictors of attention; consistent with the theoretical expectation that external technological stimuli are the primary correlates of the extraneous load that disrupts focus.

#### 5.2.2 Self‐Regulation, Coping, and the Primacy of Emotional State

The findings for behavioral impulsivity and, to a lesser degree, cognitive load align with an integrated account drawing on three frameworks. The dual‐process model distinguishes a fast, impulsive System 1 from a slow, deliberate System 2 that governs self‐control. Self‐regulation theory frames this control capacity as a finite resource that can be depleted by stress and emotional demands (ego depletion), leaving behavior more likely to default to immediate gratification. Compensatory Internet Use Theory adds that negative emotional states often prompt people to turn to digital media for relief. Read together, these frameworks suggest that problematic digital behavior may, for some individuals, represent a downstream correlate of a depleted emotional state rather than its origin.

Consistent with this, the strongest predictors of these outcomes were not tech habits alone but lifestyle factors reflecting emotional state. Q21 (“I have experienced symptoms of anxiety or depression in the past year”) was the top predictor of behavioral impulsivity in all four models and a leading predictor of cognitive load, with Q24 (emotional instability) and Q20 (stress) also prominent for both. In associational terms, this is consistent with the ego‐depletion account: self‐reported emotional distress is the predictor most strongly linked to these outcomes, and under such depletion, impulsive, reward‐seeking responses to digital cues are theorized to become more likely.

The pattern across tech‐specific items is consistent with how this vulnerability may be expressed through technology. The high importance of Q2 (“I often scroll… longer than I originally intended”) for both cognitive load and impulsivity fits the interpretation of unintended scrolling as a self‐regulation failure, and its co‐occurrence with distress (Q21) and escape‐motivated use (Q12, “I use social media as a way to escape from stress…”) is consistent with compensatory internet use. In sum, the data are consistent, though they cannot confirm a coherent associational pattern in which a depleted emotional state co‐occurs with compensatory media use and self‐regulation failures such as unintended scrolling, which together are associated with higher behavioral impulsivity. The practical implication is that impulsivity in a digital context is unlikely to be understood from digital behavior alone without also considering the user's emotional state.

This associational pattern aligns with recent qualitative findings on the “brain rot” phenomenon, in which university students report that continuous exposure to low‐quality digital media contributes to subjective mental fog, emotional desensitization, and impaired decision‐making (Özbay 2026). The pattern of continuous, unguided scrolling captured by Q2 also exemplifies the construct of “digital obesity,” recently quantified as a multidimensional behavioral excess driven by largely unconscious digital habits that strain cognitive resources (Özbay et al. 2025).

### 5.3 Implications for Digital Well‐Being and Interventions

The findings of this study have important practical implications for designing mental health and digital well‐being interventions, especially for young adults.

The results suggest that general advice to simply “reduce screen time” is not enough. The key predictors in this research highlight the need for more specific, behavior‐focused strategies. For example, the strong predictive role of Q2 (unintended scrolling) and Q15 (difficulty refocusing) for attention indicates that interventions could aim to develop metacognitive skills. This means teaching mindful disengagement from platforms and helping individuals recognize the “cognitive costs” of task‐switching, rather than relying on time limits alone.

This need for metacognitive development aligns with recent qualitative findings showing that students attempting to mitigate digitally‐associated cognitive fatigue rely on conscious self‐regulation strategies, including mindfulness, physical activity, and structured digital detoxes (Özbay 2026). Institutional interventions could therefore facilitate the conscious limitation of technology to restore digital balance, potentially using newly validated, multidimensional instruments such as the Digital Detox Scale (Akdeniz Kudubeş et al. 2025) and the Digital Obesity Scale (Özbay et al. 2025) to systematically assess and address these largely unconscious usage patterns.

Additionally, the strong predictive role of Q21 (symptoms of anxiety/depression) for cognitive load and impulsivity is a critical finding. It suggests that, for many people, problematic digital behavior may be better understood as a correlate or downstream marker of deeper emotional distress rather than the primary problem. This emphasizes the need for integrated interventions. A digital detox or a time‐management app may have limited effect if the factors associated with excess media use such as anxiety, stress, or depression are not addressed through appropriate mental health support.

Finally, the strong predictive power of the models, which rely completely on easy‐to‐access self‐report data, shows that it's possible to develop behavior‐focused screening tools. These tools could be used broadly in universities or clinical settings to identify individuals at risk for attention problems or impulsivity, enabling timely and specific support before these issues worsen.

### 5.4 Strengths and Limitations

This study has several key strengths that support its findings, along with limitations that should be considered when interpreting the results.

#### 5.4.1 Strengths

The main strength of this study is its rigorous analytical approach. A 5 × 5 nested cross‐validation procedure was used to provide unbiased estimates of model performance. This technique is resistant to the optimistic bias often present in standard cross‐validation.

By examining four different ML algorithms (KNN, SVR, RF, and XGBoost), the study ensures that its findings are not influenced by the biases or limitations of a single model.

The use of two different methods for determining feature importance—permutation importance from the unbiased nested CV and SHAP on the final refit models—offers a strong, cross‐validated agreement on the most important predictors.

The outcome scales were further examined through CFA, which supported their composite reliability and confirmed that the three constructs are empirically distinguishable rather than a single undifferentiated dimension.

The study uniquely aims to ground its predictive model in established psychological theory. By applying concepts from various frameworks such as self‐regulation, cognitive load, and compensatory use, the research goes beyond basic pattern detection to create a clear, data‐driven narrative that is interpreted in light of the links between digital habits and cognitive outcomes.

#### 5.4.2 Limitations

The study depends entirely on self‐reported data for both the predictors (digital habits, lifestyle) and the outcomes (attention, cognitive load, impulsivity), and all variables were measured with the same instrument at a single time point. This design is susceptible to common method bias, as well as to recall error and social‐desirability effects. Importantly, shared method variance can inflate the observed associations between predictors and outcomes; the predictive performance reported here (e.g., the *R* ^2^ values) may therefore be optimistic relative to what would be obtained were the outcomes measured through objective or behavioral means. The reported estimates should accordingly be interpreted as upper‐bound indicators of predictive strength under a common‐method design.

Also, the study's cross‐sectional design means that, while it identifies strong predictive relationships, it cannot establish causality. For example, it is unclear whether specific digital behaviors contribute to cognitive difficulties or whether individuals with preexisting cognitive difficulties are more likely to engage in those behaviors; the associations are equally consistent with bidirectional influence.

The sample consisted predominantly of young‐adult university students in Bangladesh, with a small number of older respondents. Beyond the usual constraints on age‐related generalization, this sampling frame carries cultural and contextual specificity that may shape the observed relationships. Patterns of digital engagement, the social meaning of online validation, norms surrounding academic pressure, and prevailing connectivity and platform ecosystems differ across cultural settings, and the strength or even direction of the distress behavior associations identified here may vary accordingly in other populations. The findings should thus be regarded as specific to this demographic and cultural context until replicated in more diverse and cross‐cultural samples.

Finally, although the outcome scales demonstrated strong composite reliability, the CFA indicated that the average variance extracted fell below the conventional 0.50 threshold and that several negatively‐worded items loaded weakly. This suggests that the adapted scales, while internally consistent and structurally distinct, would benefit from further psychometric refinement in future applications.

## 6 Conclusion and Future Work

This study successfully showed that a nuanced understanding of a person's cognitive and behavioral patterns can be achieved by using ML models on their self‐reported digital and lifestyle habits. This research moved past basic measurements of screen time. It showed that certain theory‐informed behaviors strongly predict attention, cognitive load, and impulsivity. The findings highlighted an important difference in the predictors of these outcomes. Attentional deficits were mainly associated with tech‐related behaviors indicative of a failure in self‐regulation and cognitive overload. In contrast, cognitive load and impulsivity were most strongly associated with indicators of emotional and mental health.

The results are consistent with a coherent, though noncausal, theoretical pattern, given the cross‐sectional design. This pattern suggests that, for many individuals, problematic digital behavior may be less a primary cause of difficulty than a maladaptive coping response that co‐occurs with preexisting distress: A depleted internal state, marked by anxiety and stress, is associated with compensatory media use, which in turn co‐occurs with self‐regulation failures such as prolonged unintended scrolling and, ultimately, with higher behavioral impulsivity. This interaction between lifestyle factors and tech behaviors underscores a central point: Impulsivity in a digital context is unlikely to be fully understood without also considering the user's emotional state.

Future research should build on these findings by addressing the study's limitations. Longitudinal studies are needed to disentangle the causal and temporal relationships between these variables. To address the limitations of self‐reporting, future work should incorporate objective data, such as platform‐level behavioral logs and screen‐time tracking, or behavioral measures from cognitive tasks. Finally, this research should be repeated with larger, more diverse samples to test the generalizability of these predictive models across different demographic and cultural groups. The ultimate goal of this line of inquiry should be the development and empirical testing of targeted digital well‐being interventions informed by the specific behavioral mechanisms identified in this study.

## Author Contributions

**Arfatul Islam Asif**: conceptualization, methodology, software, validation, formal analysis, investigation, resources, data curation, writing – original draft, writing – review and editing, visualization, project administration. **Sanjoy Das Joy**: conceptualization, software, validation, formal analysis, investigation, resources, data curation, writing – review and editing, visualization. **Mustakim Billah**: conceptualization, software, validation, formal analysis, investigation, resources, writing – review and editing, visualization. **Unayes Ahmed Khan**: validation, formal analysis, investigation, resources, writing – review and editing, visualization. **Ibnul Mansib**: validation, formal analysis, investigation, resources, writing – review and editing, visualization. **Sheikh Md. Azizul Hoque Munna**: investigation, resources, writing – review and editing. **Anupam Kumar Bairagi**: writing – review and editing. **Abdullah Al Noman**: supervision, validation, investigation, resources, writing – review and editing.

## Funding

The authors have nothing to report.

## Ethics Statement

The study protocol was reviewed and approved by the School Ethics Review Committee (SERC) at Shahjalal University of Science and Technology (reference no: SAST/341/176, date: 09/24/2025). As part of the comprehensive review, the protocol and survey instruments were also vetted by the university‐appointed psychologist. All data were collected using a custom‐built web application. Before participating, users saw a digital informed consent form on the initial page of the application. This form explained that all data would be collected anonymously, that participation was voluntary, and that providing an email address was optional. All participants provided informed consent by agreeing to these terms before proceeding to the questionnaire.

## Conflicts of Interest

The authors declare no conflicts of interest.

## Data Availability

The data analyzed during this study are available from the corresponding author upon reasonable request. Only anonymized responses to the Likert‐scale (1–5) questionnaire can be provided; no personal and demographic information (such as optional email, gender, or age) is available.

## Complete Survey Instrument

**TABLE A1:** Complete 55‐item questionnaire covering all predictor and outcome variables.

Item	Statement	Scale
**Part A: Tech usage factors items** 1 = strongly disagree, 2 = disagree, 3 = neutral, 4 = agree, 5 = strongly agree		
Q1	I spend several hours daily watching short‐form videos on TikTok, Instagram reels, or YouTube shorts.	1 2 3 4 5
Q2	I often scroll through social media feeds longer than I originally intended.	1 2 3 4 5
Q3	I feel the urge to check my social media notifications as soon as they appear.	1 2 3 4 5
Q4	I frequently binge‐watch content on YouTube, Netflix, or other streaming platforms.	1 2 3 4 5
Q5	The autoplay feature on video platforms makes it difficult for me to stop watching.	1 2 3 4 5
Q6	I frequently use disappearing message apps like Snapchat, Instagram stories, or WhatsApp.	1 2 3 4 5
Q7	I feel anxious when my posts do not receive likes, comments, or shares quickly.	1 2 3 4 5
Q8	Social media notifications often distract me from work, studies, or real‐life activities.	1 2 3 4 5
Q9	I often refresh my social media feeds to check for new updates.	1 2 3 4 5
Q10	I check social media as soon as I wake up in the morning.	1 2 3 4 5
Q11	I feel mentally exhausted after spending a long time on social media.	1 2 3 4 5
Q12	I use social media as a way to escape from stress or negative emotions.	1 2 3 4 5
Q13	I feel pressured to maintain an online presence or follow trends on social media.	1 2 3 4 5
Q14	I feel the need to respond immediately to messages or comments on social media.	1 2 3 4 5
Q15	After using social media, I find it difficult to focus on real‐world tasks.	1 2 3 4 5
**Part B: Lifestyle and psychosocial factors items** 1 = never, 2 = rarely, 3 = sometimes, 4 = often, 5 = always		
Q16	I get at least 7–8 h of sleep on most nights (reverse scored).	1 2 3 4 5
Q17	I have trouble sleeping when I use screens before bedtime.	1 2 3 4 5
Q18	I engage in regular physical activity, such as exercise or sports (reverse scored).	1 2 3 4 5
Q19	I believe my diet and nutrition impact my mental well‐being.	1 2 3 4 5
Q20	I frequently experience high levels of stress due to academic, work, or personal pressures.	1 2 3 4 5
Q21	I have experienced symptoms of anxiety or depression in the past year.	1 2 3 4 5
Q22	Social media has affected my self‐esteem or body image.	1 2 3 4 5
Q23	I often compare myself to others based on their social media posts.	1 2 3 4 5
Q24	Over the past month, I have felt emotionally stable and in good mental health (reverse scored).	1 2 3 4 5
Q25	I feel that social media or digital interactions have negatively affected my real‐life relationships.	1 2 3 4 5
**Part C: Attention and Focus Scale items** 1 = never, 2 = rarely, 3 = sometimes, 4 = often, 5 = very often		
Q26	I have trouble keeping my attention on tasks that are boring or repetitive.	1 2 3 4 5
Q27	I often start tasks but leave them unfinished.	1 2 3 4 5
Q28	I find myself checking notifications or my phone even during important tasks.	1 2 3 4 5
Q29	I frequently lose or misplace items needed for daily tasks (e.g., keys, phone, notes).	1 2 3 4 5
Q30	I get distracted easily by sounds, activity, or movement around me.	1 2 3 4 5
Q31	My house or workspace contains many half‐finished projects.	1 2 3 4 5
Q32	When switching between apps or tasks, I find it hard to maintain mental focus.	1 2 3 4 5
Q33	I struggle to stay present and focused when using digital devices.	1 2 3 4 5
Q34	I make careless mistakes on tasks that require full attention.	1 2 3 4 5
Q35	I forget appointments or obligations even after planning for them.	1 2 3 4 5
**Part D: Cognitive Load Scale items** 1 = strongly disagree, 2 = disagree, 3 = neutral, 4 = agree, 5 = strongly agree		
Q36	I get tired very quickly when doing mentally demanding tasks.	1 2 3 4 5
Q37	I have problems thinking clearly when I'm mentally overwhelmed.	1 2 3 4 5
Q38	I tire easily after periods of concentration or problem‐solving.	1 2 3 4 5
Q39	I feel mentally exhausted even without doing physical work.	1 2 3 4 5
Q40	I have trouble starting tasks that require a lot of thought.	1 2 3 4 5
Q41	I feel rested and mentally fresh (reverse scored).	1 2 3 4 5
Q42	I find it difficult to maintain focus when switching between tasks.	1 2 3 4 5
Q43	I find that my thoughts wander when I try to concentrate.	1 2 3 4 5
Q44	I can concentrate quite well when I need to (reverse scored).	1 2 3 4 5
Q45	I experience a drop in mental clarity when I deal with too much information at once.	1 2 3 4 5
**Part E: Behavioral Impulsivity Scale items** 1 = never, 2 = rarely, 3 = sometimes, 4 = often, 5 = very often		
Q46	I do things without thinking.	1 2 3 4 5
Q47	I act on the spur of the moment.	1 2 3 4 5
Q48	I buy things impulsively, even when I don't need them.	1 2 3 4 5
Q49	I say things without thinking.	1 2 3 4 5
Q50	I get easily bored when I am working on thought problems or repetitive tasks.	1 2 3 4 5
Q51	In the last month, I felt nervous and stressed.	1 2 3 4 5
Q52	In the last month, I felt that I was unable to control important things in my life.	1 2 3 4 5
Q53	I am more interested in the present than the future.	1 2 3 4 5
Q54	In the last month, I felt difficulties were piling up so high that I couldn't overcome them.	1 2 3 4 5
Q55	I usually think carefully before making decisions (reverse scored).	1 2 3 4 5

**TABLE A2:** Best hyperparameters for final random forest models.

Hyperparameter	Attention	Cognitive load	Behavioral impulsivity
n_estimators	500	50	100
max_depth	10	6	14
min_samples_split	2	4	4
min_samples_leaf	1	1	1
max_features	“sqrt”	“sqrt”	“sqrt”

**TABLE A3:** Best hyperparameters for final KNN models.

Hyperparameter	Attention	Cognitive load	Behavioral impulsivity
n_neighbors	11	5	33

**TABLE A4:** Best hyperparameters for final SVR models.

Hyperparameter	Attention	Cognitive load	Behavioral impulsivity
C	10	10	10
epsilon	0.01	0.01	0.01
gamma	0.0001	0.0001	0.0001
kernel	“rbf”	“rbf”	“rbf”

**TABLE A5:** Best hyperparameters for final XGBoost models.

Hyperparameter	Attention	Cognitive load	Behavioral impulsivity
learning_rate	0.03	0.01	0.03
max_depth	5	3	2
n_estimators	200	300	100
subsample	0.7	0.7	0.7
